# Molecular Analysis of the Freshwater Prawn *Macrobrachium olfersii* (Decapoda, Palaemonidae) Supports the Existence of a Single Species throughout Its Distribution

**DOI:** 10.1371/journal.pone.0054698

**Published:** 2013-01-31

**Authors:** Natália Rossi, Fernando Luis Mantelatto

**Affiliations:** Laboratory of Bioecology and Crustacean Systematics (LBSC), Program in Comparative Biology, Department of Biology, Faculty of Philosophy, Science and Letters of Ribeirão Preto (FFCLRP), University of São Paulo (USP), Ribeirão Preto, São Paulo, Brazil; George Washington University, United States of America

## Abstract

*Macrobrachium olfersii* is an amphidromous freshwater prawn, widespread along the eastern coasts of the Americas. This species shows great morphological modifications during ontogenesis, and several studies have verified the existence of a wide intraspecific variation. Because of this condition, the species is often misidentified, and several synonyms have been documented. To elucidate these aspects, individuals of *M. olfersii* from different populations along its range of distribution were investigated. The taxonomic limit was established, and the degree of genetic variability of this species was described. We extracted DNA from 53 specimens of *M. olfersii*, *M. americanum*, *M. digueti* and *M. faustinum,* which resulted in 84 new sequences (22 of 16S mtDNA, 45 of Cythocrome Oxidase I (COI) mtDNA, and 17 of Histone (H3) nDNA). Sequences of three genes (single and concatenated) from these species were used in the Maximum Likelihood and Bayesian Inference phylogenetic analyses and COI sequences from *M. olfersii* were used in population analysis. The genetic variation was evaluated through the alignment of 554 bp from the 16S, 638 bp from the COI, and 338 bp from the H3. The rates of genetic divergence among populations were lower at the intraspecific level. This was confirmed by the haplotype net, which showed a continuous gene flow among populations. Although a wide distribution and high morphological intraspecific variation often suggest the existence of more than one species, genetic similarity of Caribbean and Brazilian populations of *M. olfersii* supported them as a single species.

## Introduction

Prawns of the genus *Macrobrachium* Spence Bate, 1861 are important members of freshwater and estuarine systems [1], [2]. The diversity of species is tremendous, with more than 240 species recognized worldwide [3], [4]. Some of them need estuarine and freshwater to complete their life cycle, which implicates in recurring migrations between both environments. The prawns that have this behavior are called as amphidromous and they show many larval and reproductive peculiarities [5]. In some amphidromous freshwater shrimps, the females live in freshwater and migrate downstream, near to estuaries, where the hatching occurs. After larval development in salt water, the juveniles migrate up to the freshwater [6]. *Macrobrachium olfersii* (Wiegmann, 1836) is one these species, with larval stages requiring high salinity during development [7], [8]. Therefore, it is finding along of coastland and its geographic distribution covers almost all the eastern coasts of the Americas, from the southern United States, where the prawns were introduced [9], [10], [11], to Mexico, Guatemala, Costa Rica, Panama, and Venezuela to southern Brazil [12], [9], [11], [13], [14], [15].

Species that have a wide distribution, in heterogeneous or geographically isolated environments can have a phenotype variation, because they are prone to show plastic responses to different environmental influences. Further these plasticity environment-dependent, the phenotypic variations also can stem from genetic or behavior differences between individuals, from ontogenetic developmental or combining of all these factors [16]. On the other hand, morphological characters may often be undergoing convergent evolution as they are under similar selective pressure [17]. The species of the genus *Macrobrachium* have high intraspecific variation and a single species may have genetic diversity and structured populations [18].

*Macrobrachium olfersii* is in this context, it has a wide distribution and a great morphological variation during its ontogenesis [19], [20], [21], [22], including the possibility of morphotypes within the species. This fact have already observed in other congeneric species such as *Macrobrachium rosenbergii* (de Man, 1879), *M. amazonicum* (Heller, 1862) and *M. grandimanus* [23], [24], [25]. The presence of plastic characters in the genus *Macrobrachium* makes the accurate determination of species more difficult and problematic [10], [26].

Commonly, *Macrobrachium olfersii* is confused with species that occur preferentially in Central America such as *M. faustinum* (Saussure, 1857), *M. crenulatum* Holthuis, 1950, *M. digueti* (Bouvier, 1895), *M. hancocki* Holthuis, 1950, and *M. acanthochirus* Villalobos, 1967. Because of their close morphological similarities, these species were designated as a possible species complex by Villalobos [10]. Recently, *Macrobrachium birai* Lobão, Melo & Fernandez, 1986 and *Macrobrachium holthuisi* Genofre & Lobão, 1978 were considered junior synonyms of *M. olfersii* [27], [28].

Together, this information illustrates that sometimes morphological analysis alone is not sufficient to resolve the diversity of species complexes. The addition of molecular data has proven very useful to elucidate the taxonomic relationships in morphologically variable groups of freshwater prawns [29], [2], [27], [18], [30], [31]. Molecular markers can be useful in delimiting boundaries between lineages and/or species and can help in the interpretation of biodiversity patterns [32], [33], [27], [18], [31]. The mitochondrial markers, such as 16S and Cytochrome Oxidase I (COI), have high mutation rate, which makes it very useful at intraspecific levels, but causes increasing saturation when older splits are analyzed. Therefore, the combination of mtDNA with more conserved nuclear markers, such as Histone 3 (H3), may be used to support phylogenetic conclusions [34], [35]. The H3 is considered a small gene with about 350 bp, which are very conservative and relative ease to amplify. When we combined nuclear with mitochondrial genes, we provide a broad spectrum of inference and great insights into the evolutionary history of *Macrobrachium*.

Considering that *Macrobrachium* inhabits heterogeneous environments and shows morphological dissimilarity and genetic variability within populations [18], this study examined individuals of *M. olfersii* from different populations along its distribution, in order to establish the taxonomic boundaries and to describe the degree of genetic variability.

## Materials and Methods

### Sample Collection

Some specimens of *M. olfersii* were obtained from field collections in different locations, under license from the appropriate authorities (Table 1). The collections of species conducted in this study complied with current applicable state and federal laws of Brazil (DIFAP/IBAMA 126/05; permanent license to FLM for collection of Zoological Material N^o^. 11777-1 MMA/IBAMA/SISBIO). No material were obtained from national park or other protected area of land and we confirm that the field studies did not involve endangered or protected species.

**Table 1:** Sequences of *Macrobrachium* and outgroup species used this study.

Species	Sample locality	Abbrev.	Catalogue no.	GenBank accession code		
				16S	COI	H3
***Macrobrachium americanum***	Costa Rica (Pacific)	CR-PA	CCDB 2883	JQ805797	JQ805899	JQ805861
***Macrobrachium digueti***	Mexico (Pacific)	MX-PA	CNCR 24811	–	JQ805905	–
***Macrobrachium digueti***	Costa Rica (Pacific)	CR-PA	CCDB 2882	JQ805806	JQ805903	JQ805868
***Macrobrachium digueti***	Costa Rica (Pacific)	CR-PA	CCDB 3091	JQ805807	JQ805904	JQ805869
***Macrobrachium faustinum***	Jamaica (northeast)	JM-NE	RMNHD 17613	JQ805809	JQ805907	–
***Macrobrachium lar***	N/A	–	GUMB 992	**EF588316**	–	**EU249462**
***Macrobrachium lar***	China (Taiwan)	CH-TW	–	–	**AB235270**	–
***Macrobrachium olfersii***	Mexico	MX	–	**AY377849**	–	–
***Macrobrachium olfersii***	Costa Rica (Atlantic)	CR-AT	CCDB 2876	JQ805835	JQ805933	JQ805887
***Macrobrachium olfersii***	Costa Rica (Atlantic)	CR-AT	CCDB 2874	JQ805838	JQ805935	JQ805889
					JQ805957	
					JQ805958	
***Macrobrachium olfersii***	Costa Rica (Atlantic)	CR-AT	CCDB 2880	JQ805839	JQ805936	JQ805890
***Macrobrachium olfersii***	Costa Rica (Atlantic)	CR-AT	CCDB 3754	–	JQ805938	–
***Macrobrachium olfersii***	Panama (Atlantic)	PN-PA	CCDB 2884	JQ805840	JQ805937	JQ805891
					JQ805959	
					JQ805960	
***Macrobrachium olfersii***	Venezuela (northwest)	VZ-NW	IVIC 1083	JQ805837	JQ805934	JQ805888
***Macrobrachium olfersii***	Venezuela (Isla Margarita)	VZ-IM	CCDB 2446	JQ805836	JQ805932	JQ805886
***Macrobrachium olfersi***	Brazil (Bahia)	BR-BA	CCDB 2720	JQ805833	JQ805930	–
					JQ805956	
***Macrobrachium olfersii***	Brazil (Bahia)	BR-BA	CCDB 2439	JQ805827	JQ805924	JQ805881
***Macrobrachium olfersii***	Brazil (Espírito Santo)	BR-ES	CCDB 3084	JQ805826	JQ805923	JQ805880
***Macrobrachium olfersii***	Brazil (Espírito Santo)	BR-ES	CCDB 3082	JQ805832	JQ805929	JQ805885
					JQ805955	
***Macrobrachium olfersii***	Brazil (Rio de Janeiro)	BR-RJ	CCDB 2213	JQ805829	JQ805926	JQ805882
					JQ805952	
					JQ805953	
					JQ805954	
***Macrobrachium olfersii***	Brazil (northern São Paulo)	BR-SPn	CCDB 1851	JQ805834	–	–
***Macrobrachium olfersii***	Brazil (northern São Paulo)	BR-SPn	CCDB 2423	JQ805828	JQ805925	–
					JQ805949	
					JQ805950	
					JQ805951	
***Macrobrachium olfersii***	Brazil (southern São Paulo)	BR-SPs	CCDB 2503	–	JQ805931	–
					JQ805946	
					JQ805947	
					JQ805948	
***Macrobrachium olfersii***	Brazil (Paraná)	BR-PR	CCDB 2445	JQ805830	JQ805927	JQ805883
***Macrobrachium olfersii***	Brazil (Paraná)	BR-PR	CCDB 2279	JQ805824	JQ805921	JQ805878
					JQ805943	
					JQ805944	
					JQ805945	
***Macrobrachium olfersii***	Brazil (Santa Catarina)	BR-SC	CCDB 1929	JQ805831	JQ805928	JQ805884
***Macrobrachium olfersii***	Brazil (Santa Catarina)	BR-SC	CCDB 1924	JQ805825	JQ805922	JQ805879
					JQ805939	
					JQ805940	
					JQ805941	
					JQ805941	
					JQ805942	
***Cryphiops caementarius***	–	–	JC 1219	**DQ079711**	–	**DQ079672**
***Cryphiops caementarius***	Chile (Coquimbo)	CL-CO	CCDB 1870	–	**HM352495**	–

Sample localities, locality abbreviations (Abbrev.), catalogue numbers, and GenBank accession codes are provided. The codes of sequences that were retrieved from GenBank are bold type.

Museum/collection abbreviations: CCDB: Crustacean Collection of the Department of Biology, Faculty of Philosophy, Sciences and Letters of Ribeirão Preto, University of São Paulo; MZUSP: Museu de Zoologia da Universidade de São Paulo; CNCR: Coleción Nacional de Crustaceos de la Universidad Nacional Autónoma de México; IVIC: Instituto Venezolano de Investigaciones Científicas; UCR: Museo de Zoología de la Universidad de Costa Rica; RMNHD: National Museum of Natural History, Leiden, The Netherlands.

We have collected about six individuals per site from coastal drainage of Costa Rica, Panama and different places from Brazil (States of Bahia, Espírito Santo, Rio de Janeiro, São Paulo, Paraná and Santa Catarina) (Table 1), by sieving amongst marginal vegetation and under the rocky bottom of rivers and streams. After sampling, individuals were separated, immediately preserved in ethanol (80%) and deposited in Crustacean Collection of the Biology Department (CCDB) of the Faculty of Philosophy, Sciences and Letters of Ribeirão Preto (FFCLRP) at the University of São Paulo (USP) (Permanent license for Crustacean Collection N^o^. 071/2012/SECEX/CGEN).

Additional material was also obtained via donation or loan from the following museums and crustacean collections: Museum of Zoology, University of São Paulo, São Paulo, Brazil (MZUSP); Coleccíon Nacional de Crustáceos de La Universidad Nacional Autonoma de Mexico, Mexico City, Mexico (CNCR); Instituto Venezolano de Investigações Científicas, Venezuela (IVIC); Museum of Zoology, University of Costa Rica (MZUCR), Rijksmuseum Van Natuurlijke Historie, Leiden, Holland (RMNH) and (Table 1).

By these means of sampling (field collection, donation or loan of species from museums and crustacean collection), we were able to include in our analysis specimens from almost the entire range of distribution of *M. olfersii,* in order to have the most robust possible data set. Unfortunately, species from United States and Guatemala were not sampled. Nevertheless, our sampling effort was enough to analyze the variability of *M. olfersii* due to wide distribution this species.

*Macrobrachium faustinum* and *M. digueti*, which belong to the supposed *M. olfersii* complex [10], were included in our analysis because they are closest species to *M. olfersii*. *Macrobrachium americanum* Spence Bate, 1868, from Pacific coast of South and Central America, *Macrobrachium lar* (Fabricius, 1798) from Indo-Pacific and *Cryphiops caementarius* (Molina, 1782) from Pacific coast of South America were added. Previous phylogenetic analyses showed that they are closely related to species of the *M. olfersii* complex [36], [32], [37], [27] (Table 1).

*Macrobrachium americanum* and *M. lar* have similar life histories with extended larval development, and similar and diverse geographic distributions. Although *C. caementarius* belong to a different genus, it has been positioned among species of *Macrobrachium* [27]. This questionable phylogenetic position have shown a closely relationship with *M. olfersii* complex [27]. Like the sampling methods of the *M. olfersii* specimens, the exemplars of the *M. faustinum*, *M. digueti* and *M. americanum* were obtained by field collecting from Costa Rica and Panama, others exemplars from Mexico, Jamaica and Venezuela were loaned of the different museums and crustacean collections (described at Table 1).

### Species Identification

Considering the previously reported taxonomic doubts of *Macrobrachium olfersii* complex of species, a detailed comparative study of external morphology of the group (*M. acanthochirus*, *M. crenulatum*, *M. digueti*, *M. faustinum*, *M. hancocki* and *M. olfersii*) was also conducted, and a key for identification of male adults of the *M. olfersii* species-complex will be proposed based on morphological analysis of greater pereiopod (data not shown here). In order to better understand the interspecies differences in morphological characters and provide us a robust support during specimen identification and molecular analysis (Rossi & Mantelatto, in preparation). The identifications were based on the diagnostic morphological traits of *M. olfersii* and related species, in accordance with the literature [9], [10], [38], [15].

Some characteristics that differentiate the species of *M. olfersii* complex are based mainly on shape, ornament and morphometric ratio of the articles of the second pereiopod, such as the number and distribution of tooth on the upper and cutting edge of the fingers, the format of the lower margin of the palm and the ratios obtained from the carpus length/merus length. On second pereiopod of the *M. olfersii*, there are tooth on all upper margin and the lower margin is convex. The carpus is equal or smaller than merus [9,10; Rossi & Mantelatto, in preparation]. However, these small variations can often be different stages of the development or stem as phenotypic plasticity [39], [10].

### DNA Extraction, Amplification and Sequencing

**Figure 1:**
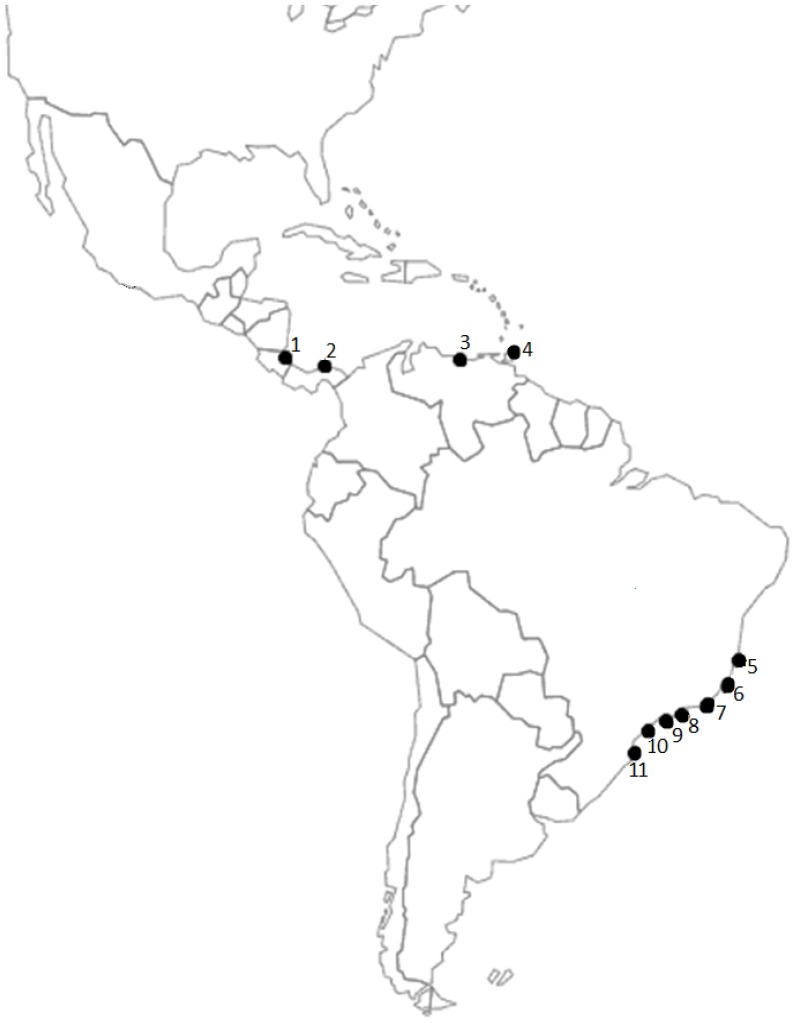
Locations of *Macrobrachium olfersii* samples used to extract DNA. 1: CR-AT; 2: PN-AT; 3: VZ-NW; 4: VZ-IM; 5: BR-BA; 6: BR-ES; 7: BR-RJ; 8: BR-SPn; 9: BR-SPs; 10: BR-PR; 11: BR-SC. Abbreviations: see Table 1.

Most sequences obtained in this study were generated from our own extractions for this project. These sequences were deposited to Genbank under the accession numbers listed in Table 1. Seven additional comparative sequences from *Macrobrachium lar*, *M. olfersii* and *Cryphiops caementarius* were retrieved from GenBank. Genetic vouchers were deposited in appropriate scientific zoological collections. DNA was extracted from abdominal or pereiopod muscle tissue from 53 *Macrobrachium* specimens from different localities (Table 1 and Fig. 1). When possible, the sequences were obtained from multiple representatives from each collection site.

DNA extraction and amplification procedures followed Mantelatto *et al.* [40], [41], [42] and Pileggi and Mantelatto [27]. Extracted tissues was macerated and incubated for 12–24 hs in 600 ml of lysis buffer at 65°C. Protein was separated with the addition of 200 ml of ammonium acetate (7.5 M), followed by centrifugation. The addition of 600 ml of cold (−20°C) absolute isopropanol was used for DNA precipitation, also followed by centrifugation. The resulting pellet was then washed with 70% ethanol, dried in a lyophilizer and resuspended in 20 ml of TE buffer.

DNA was amplified by polymerase chain reaction (PCR) using previously tested primers [43], [44]: H3ar (ATA TCC TTR GGC ATR ATR GTG AC) and H3af (ATG GCT CGT ACC AAG CAG ACV GC) for the Histone (H3) gene [45], H2 (AGA TAG AAA CCA ACC TGG) and L2 (TGC CTG TTT ATC AAA AAC AT) for the 16S gene [46], and COIa (AGT ATA AGC GTC TGG GTA GTC) and COIf (CCT GCA GGA GGA GGA GAC CC) for the Cytochrome Oxidase I (COI) gene [47].

Reactions were performed in 25 µl volumes containing 6.5 µl of distilled water, 3 µl of 10X PCR buffer II, 3 µl of MgCl_2_ (25 mM), 5 µl of betaine (5 M), 1 µl of each primer (10 mM), 4 µl of dNTP (10 mM), 0.5 µl of AmpliTaq DNA polymerase and1 µl of DNA. Thermal cycling was performed as follows for COI: initial denaturation for 2 min at 94°C, followed by 30 cycles of 30 s at 94°C, 30 s at 50°C, and 60 s at 72°C, with a final extension of 6 min at 72°C. Thermal cycling for 16S and H3∶5 min at 98°C, followed by 40 cycles of 45 s at 98°C, 45 s at 48°C, and 45 s at 72°C, with a final extension of 8 min at 72°C. The results of PCRs were looked at electrophoresis with agarose gel (1%).

PCR products were purified using the SureClean Plus kit, and sequenced with the ABI Big Dye® Terminator Mix in an ABI Prism 3100 Genetic Analyzer® following Applied Biosystems protocols. All sequences were confirmed by sequencing both strands. The consensus sequence for the two strands was obtained using BioEdit Version 7.0.7.1 [48]. Sequences were aligned in Clustal W with interface in BioEdit, with the following parameters: gap opening 10 and gap extending 0.2 [49]. Primer and indeterminate regions in beginning of the sequences were cut. The absence of stop codons in COI sequences was checked using the software BioEdit and the invertebrate codon table implemented in Mega 4 [50] in order to confirm the nonexistence of pseudogenes [51]. Apart from that, the consensus sequences were blast on Genbank and compared with our previous sequences.

### Phylogenetic Analyses

All analyses were based on a partial fragment of the 16S mtDNA, COI mtDNA and H3 nDNA genes. The phylogenetic reconstructions were built by the Maximum Likelihood (ML) method in PAUP version 4.0 beta 10 [52]. The appropriate model of evolution was previously selected under the Akaike Information Criterion (AIC) [53] obtained from the jModelTest program [54]. Heuristic searches were used for ML analyses with 100,000 replicates of random sequence additions, and nonparametric bootstrapping consisted of 100 replications [55] with 10 random sequence additions in PAUP. Only values >50% were reported. In order to estimate intra- and interspecific divergence rates, genetic distances were also calculated in PAUP using the uncorrected p distance.

Moreover, phylogenetic hypothesis were also generated by Bayesian Inference - BI in the MrBayes 3.1 program [56] for each gene data and for concatenated genes (three genes and mitochondrial). Bayesian analysis was configured to use the following parameters: sampling frequency 500, four-chain heating (three heated and one cold), and the value of Stop heating chains less than 0.01 after at least 2.5 millions of generations. Subsequently, data were collected from stationary phase and chain the initial states discarded (burning = 15%). The levels of branch support were obtained by the method of posterior probability. Trees generated from both analyzes were saved and edited by Figtree program v.1.3.1 [57].

The haplotype number of the COI sequences from at least three individuals of each studied population of the *M. olfersii* was calculated in DnaSP Version 4.10.9 [58]. The haplotype and nucleotide diversities were calculated for each population using Arlequin Version 3.1 [59]. Haplotype networks were constructed by the statistical parsimony method in TCS (Version 1.21) [60] and by the Median-Joining method in Network [61], with data preparation in DnaSP. Series of analyses of molecular variance (AMOVA) [62] were computed in Arlequin to examine the distribution of genetic variation. Analyses were run based on haplotype frequencies with no hierarchical structure (all populations in a single group) and with a subdivision between Caribbean and Brazilian populations of *M. olfersii*. The significance was tested using a nonparametric permutation procedure [62], incorporating 10,000 permutations.

## Results

In total, 22 sequences with 564 bp of 16S mtDNA, 45 sequences with 638 bp of COI without pseudogenes, and 17 sequences with 338 bp of H3 were generated. The frequencies of bases for each gene and the optimal models selected under AIC to use on Maximum Likelihood analyses (Table 2). The utilized model on Bayesian analysis was GTR for all data set.

**Table 2:** The frequencies of bases of the analyzing genes and the best-fit models selected under Akaike Information Criterion (AIC) on jModeltest for Maximum Likelihood (ML) analysis.

Gene	Adenine	Cytosine		Guanine		Thymine			Model selected	Invariable sites	Variable sites
**16S**	0.36		0.23		0.12		0.27	HKY		–	0.26
**COI**	0.25		0.12		0.30		0.31	TIM		0.63	4.41
**H3**	0.20		0.32		0.28		0.18	TrN		0.76	–

Abbreviations: HKY = Hasegawa, Kishino, Yano 85; Transitional model = TIM; Tamura-Nei = TrN.

**Figure 2:**
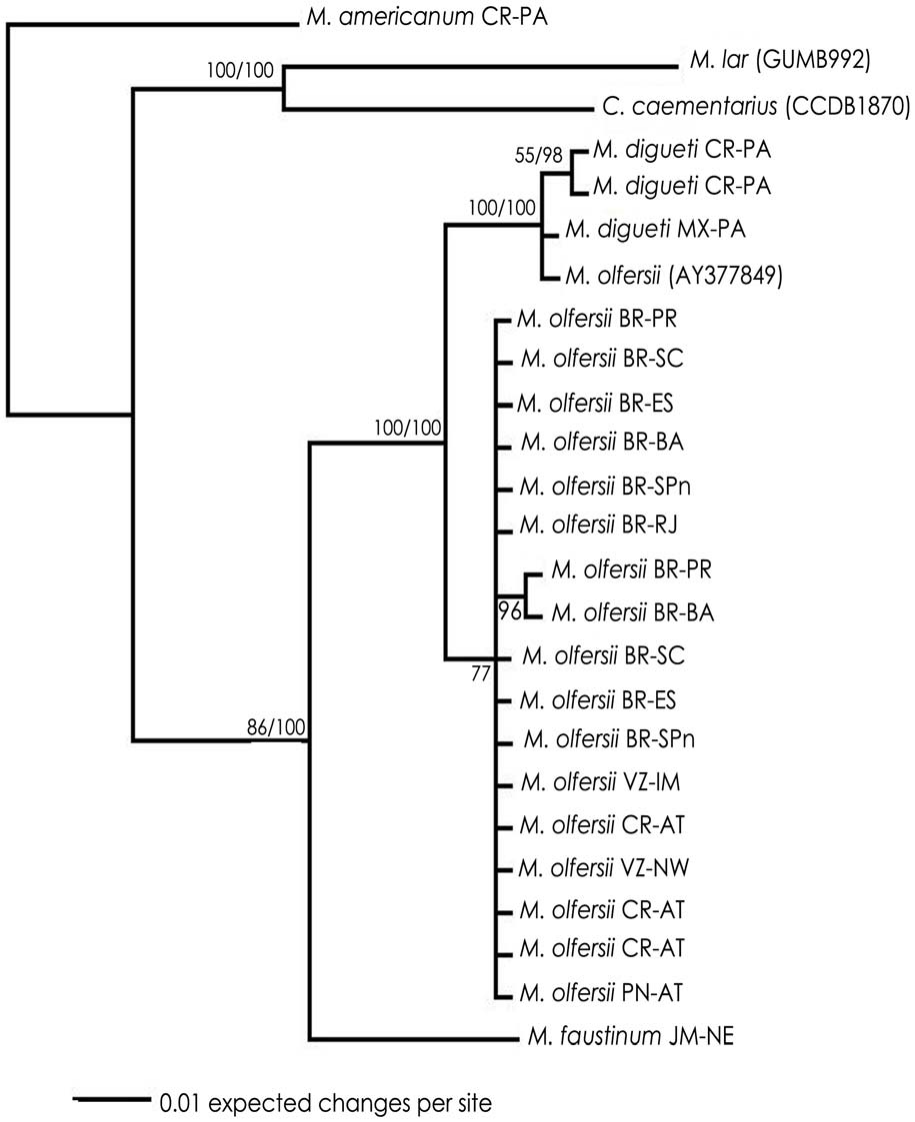
Phylogenetic tree for populations of *Macrobrachium olfersii,* based on Bayesian Inference analysis of 16S data sets. Abbreviations and *code*: see Table 1. Numbers on right: posterior probabilities; Numbers on left: bootstrap obtained on Maximum Likelihood analysis.

**Figure 3:**
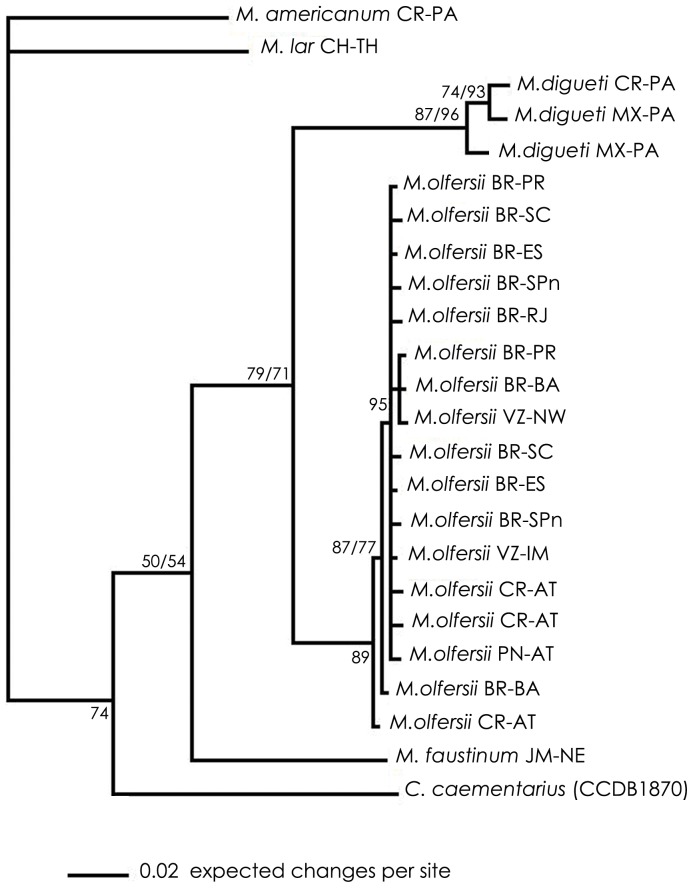
Phylogenetic tree for populations of *Macrobrachium olfersii,* based on Bayesian Inference analysis of COI data sets. Abbreviations and *code*: see Table 1. Numbers on right: posterior probabilities; Numbers on left: bootstrap obtained on Maximum Likelihood analysis.

**Figure 4:**
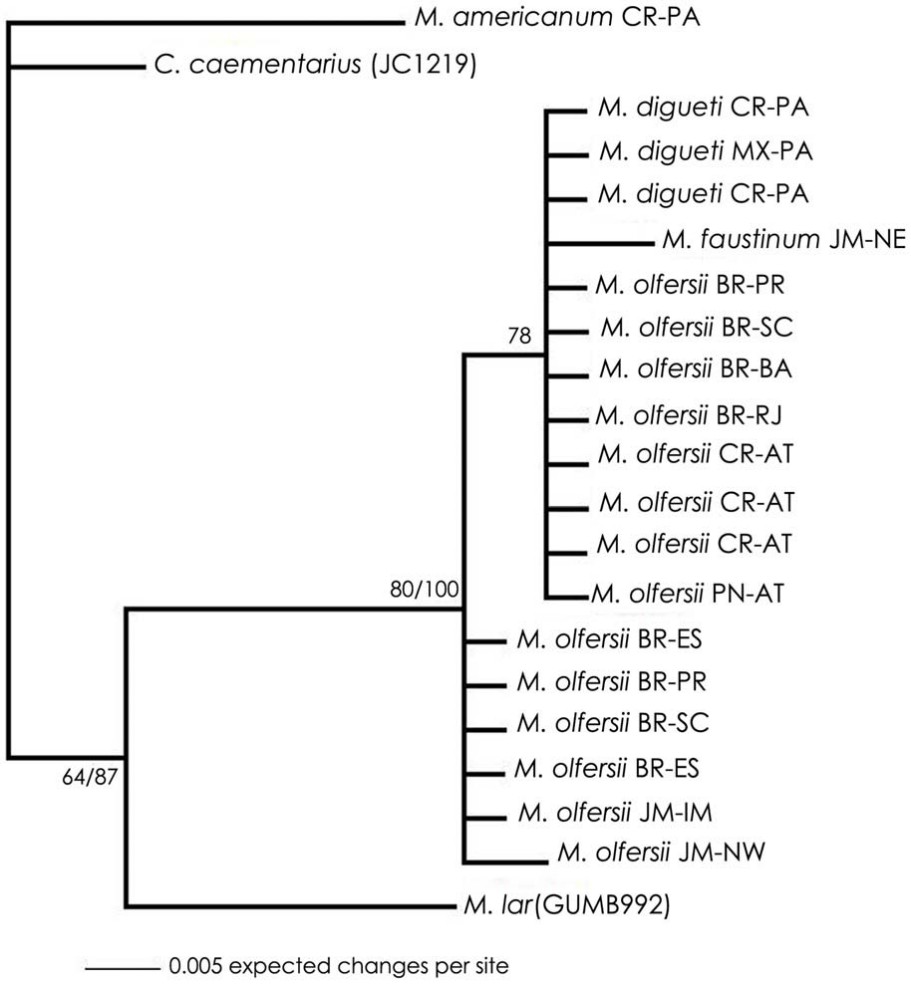
Phylogenetic tree for populations of *Macrobrachium olfersii,* based on Bayesian Inference analysis of H3 data sets. Abbreviations and *code*: see Table 1. Numbers on right: posterior probabilities; Numbers on left: bootstrap obtained on Maximum Likelihood analysis.

**Figure 5:**
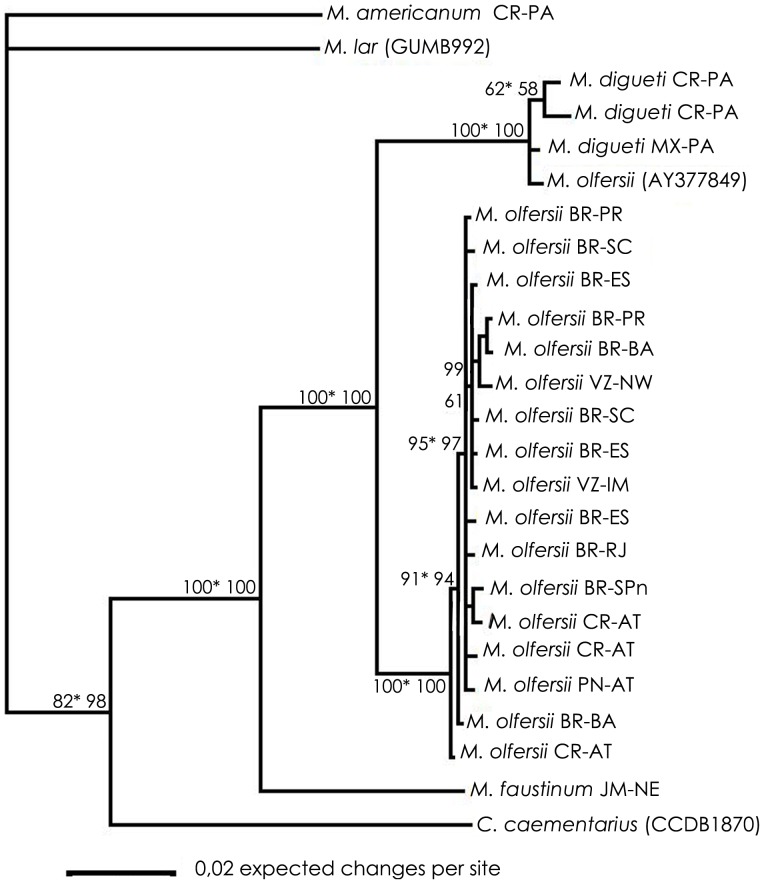
Phylogenetic tree for populations of *Macrobrachium olfersii,* based on Bayesian Inference analysis of three concatenated genes (16S, COI and H3). Abbreviations and *code*: see Table 1. Numbers: posterior probabilities; Asterisk numbers: posterior probabilities obtained by Bayesian Inference analysis of mitochondrial genes (16S and COI concatenated).

Similar topologies were achieved on ML and BI analyses for each gene datasets. Therefore, the trees obtained by BI with posterior probability were revealed and the bootstrap obtained by ML were added (Figs. 2, 3, 4, 5). Disregarding the partial sequence of 16S from *M. olfersii* that was retrieved from GenBank (ID: AY377849), phylogenetic analyses (Figs. 2, 3 and 5) show an evident separation clade from all studied species, but in nuclear, H3 (Fig. 4). Although, the species were not separated in all cases, on BI analysis based on three concatenated genes, we can see a better resolution of the branches and the same topology was obtained on mitochondrial concatenated genes (Fig. 5). Moreover, *M. olfersii* and *M. digueti* formed a sister clade with *M. faustinum* in the phylogenetic tree of 16S, COI and concatenated genes.

In general, distance analyses showed the percentage of intraspecific was lower than interspecific variation. *M. olfersii* data ranged from 0.00 to 0.18% for 16S, 0.00 to 0.95% for COI, and 0.00 to 0.27% for H3. The obtained values between *M. olfersii* and close species were: *M. digueti*, 1.25% for 16S, ranging from 10.18 to 10.72% for COI and from 0.00 to 0.27% for H3; *M. faustinum*, 3.84% for 16S, ranging from 12.51 to 12.70% for COI and from 0.27 to 0.54% for H3. Among other *Macrobrachium* species, the result was higher than before, e.g., *M. americanum* was 7.70% for 16S, ranging from 17.42 to 17.81% for COI and ranging from 5.92 to 6.26% for H3.

Based on a COI fragment of unambiguous sequence, 28 haplotypes (H) were recognized, which showed a total haplotype diversity of 0.94. Among the haplotypes, 25 (89.28%) represented single individuals, and 3 (10.72%) were polymorphic. The frequencies of haplotypes in different localities were heterogeneous (Table 3). The first haplotype (H1) was shared among three individuals from Paraná (BR-PR), two individuals from northern São Paulo (BR-SPn), one individual from Espírito Santo (BR-ES), one individual from Santa Catarina (BR-SC), one individual from Costa Rica (CR-AT), and one individual from Panama (PN-AT). The third haplotype (H3) was shared between one individual from BR-SPn and one from Costa Rica (CR-AT). The fourth (H4) was shared among one individual from BR-SC, one from BR-SPs, one individual from Bahia (BR-BA), and another from BR-RJ.

**Table 3:** Distribution of haplotypes detected in *Macrobrachium olfersii* from different localities.

Locality	Haplotypes	N	Hd±Sd
**CR-AT**	H1,H4, H11, H12, H13, H14	6	1.00±0.09
**PN-AT**	H1, H15, H25	3	1.00±0.27
**VZ-NW**	H10, H28	2	1.00±0.50
**BR-BA**	H3, H8, H24	3	1.00±0.27
**BR-ES**	H1, H7, H23	3	1.00±0.27
**BR-RJ**	H3, H5, H22	3	1.00±0.27
**BR-SPn**	H1, H4, H21, H26, H27	6	0.93±0.12
**BR-SPs**	H3, H9, H19, H20	4	1.00±0.17
**BR-PR**	H1, H18	4	0.50±0.26
**BR-SC**	H1, H2, H3, H6, H16, H17	6	1.00±0.09

Abbreviations locality: see Table 1. N: number of individuals analyzed in each population. Hd: haplotype diversity. Sd: standard deviation.

**Figure 6:**
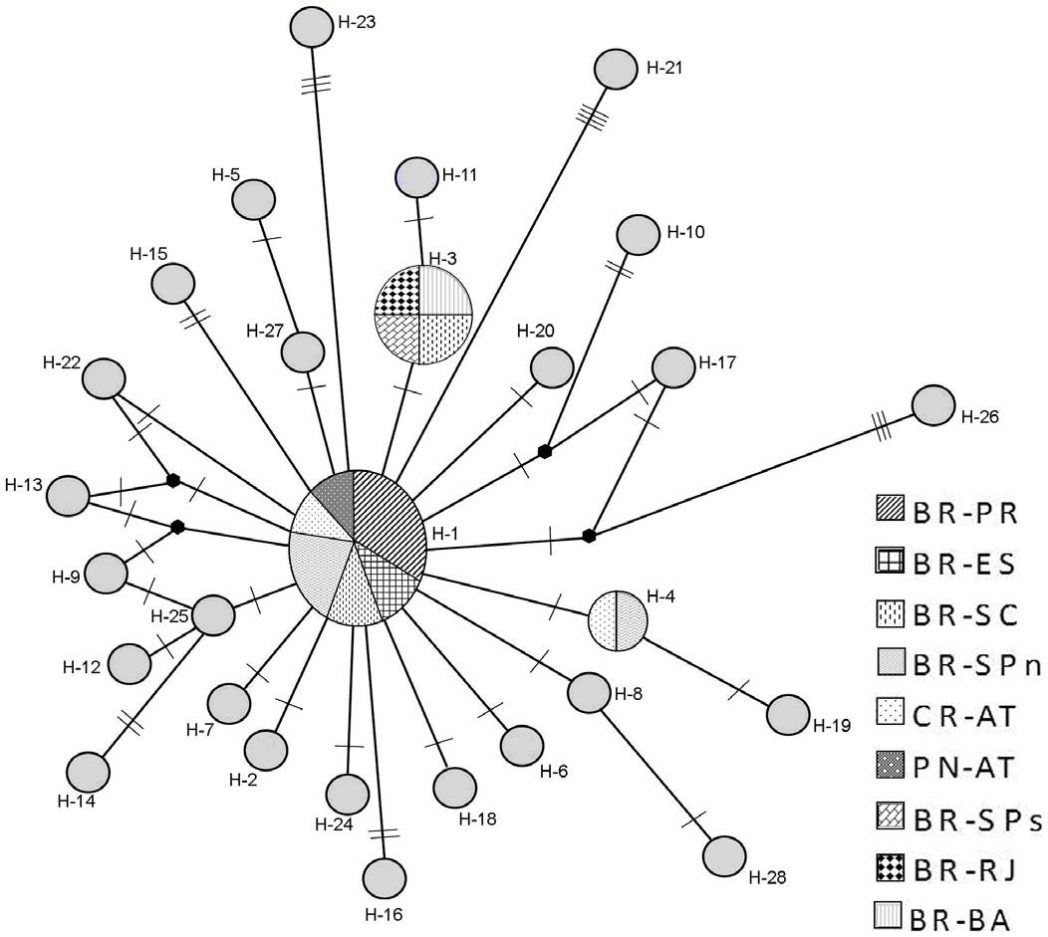
Haplotype network based on Median-Joining analysis, indicating the distribution of each haplotype found in *Macrobrachium olfersii*. The haplotype identification is below each circle. Each small trace represents a mutational step. Lines between circles indicate single-substitution differences between haplotypes (the small symbol indicates one missing haplotype inferred for two sequences that differed by two substitutions), and are not proportional to the genetic distance between haplotypes. Pattern coding indicates the origin and the frequency of the shared haplotype, indicated in the legend at right. Abbreviations: see Table 1.

Network haplotypes constructed based on the statistical parsimony method (data not shown) were equal to network haplotypes constructed based on the Median-Joining method (Fig. 6). Analysis of molecular variance (AMOVA) indicated that specimens within the *M. olfersii* population have the highest percentage of variation (98.25% without hierarchical structure and 98.18% with hierarchical structure), whereas the variation among populations was low (1.75% without hierarchical structure and 1.69% with hierarchical structure). When populations were structured according to Caribbean and Brazilian populations, the variations among groups were very low (0.13%). However the values obtained by AMOVA based on haplotype frequencies with and without hierarchical structure were not significant (Table 4).

**Table 4:** Analysis of molecular variance in *Macrobrachium olfersii*.

Structure	Source of variation	Percentage	Fixation indices	P
**Single group**	among populations	1.75	FST: 0.017	0.235
	within populations	98.25		
**Caribbean Vs. Brazilian**	among groups	0.13	FCT: 0.001	0.356
	among populations	1.69	FSC: 0.016	0.235
	within populations	98.18	FST: 0.018	0.256

Values were obtained with no hierarchical structure (all populations in a single group) and with a subdivision between Caribbean and Brazilian populations. The Caribbean group included specimens from Costa Rica, Panama and Venezuela. The Brazilian group included specimens from Bahia, Espírito Santo, Rio de Janeiro, São Paulo, Paraná and Santa Catarina.

## Discussion

Our molecular results confirm the taxonomic status of *M. olfersii* as a valid species by analysis of individuals from Caribbean and Brazilian regions and a close phylogenetic relationship with other related species. Despite the absence of all species within the complex, we chose the closest species (*M. digueti* and *M. faustinum*), which really could be synonymous from *M. olfersii*. As far as we know, after about 43 years since the proposal of a possible morphological species complex, this is the most thorough study using the molecular phylogeny approach to elucidate the status of this species. Different analyses were computed and compared. This methodology had given consistent results, because we can see similar topologies and inferences.

Maximum likelihood (ML) has been considered a good reconstruction method in studies with Decapods [63], [27], [18], [64], [31]. These results were compared with obtained by Bayesian Inference (BI) that has also been efficient [65], [63], [66]. Similar topologies were achieved, although BI showed better resolution of the branches with high posterior probabilities.

We opted by a phylogenetic approach, firstly because of we followed the Phylogenetic Species Concepts sensu Mishler & Theriot [67] as well this methodology spans intraspecific and interspecific evolution and provides evidence fundamental to inferring the process of speciation [68], [69]. Others species delimitation methods could be analyzed, such as [17], [69]. However, we did diverse analyses and obtained consistent results.

Distance analyses showed that the intraspecific genetic variation within *M. olfersii* is lower than the interspecific variation [27]. This divergence is strongly based on three genes; however, the COI gene shows the most difference among the closest species, corroborating the utility of the COI gene as a good marker to separate close and sibling species. Moreover, within the *M. olfersii* clade illustrated in all phylogenetic analyses, there is no genetic structure and no haplotype fixed in a single population. Therefore, the observed morphological variability must be phenotypic plasticity [10]. In the haplotype network, this condition is supported by haplotype sharing (polymorphic), showing a continuous gene flow among Caribbean and Brazilian populations.

### *M. olfersii×M. digueti*

From the COI datasets, we found that *M. olfersii* remains in a single clade in ML and BI analyses (Fig. 3). However, analyses based on 16S datasets (Fig. 2) and on concatenated genes, the sequence of *M. olfersii* retrieved from GenBank (ID: AY377849) was not separated from *M. digueti* branch. Although this specimen had been not analyzed, these results suggest that the identification this exemplar is incorrect. On H3 datasets indicated that close congener species appeared inside the *M. olfersii* clade (Fig. 4). Besides, this intimate relationship would be related to the condition of sibling species between *M. olfersii* and *M. digueti* [9].

*Macrobrachium olfersii* occurs on Atlantic and *M. digueti* occurs on Pacific coast of Americas. They are closely related and there are a small number of differences morphological between them. Apart from the condition of sibling species, they are considered cryptic species (Rossi & Mantelatto, in preparation). Their geographic distribution, the obtained divergence by COI and 16S, the phylogenies based on COI analysis confirm the valid of both species. The results by H3 showed the high genetic similarities, due to be more conservative gene. Although we did not clock molecular analysis, we suggested that the split of these species could be associated with closure of the Isthmus of Panama due these genes have lower rates of evolution and the pattern of the geographic distribution.

It is possible that these taxa have radiated following the closure of the Isthmus of Panama, similarly to other decapods such as the snapping shrimps [70]. In contrast, some studies have indicated that some cryptic species-complexes of shrimps show older (pre-Isthmian) divergences that were probably responses to environmental changes [71], [72], [73]. Consequently, these closely related sibling species cannot be separated by using only conservative genes. The results by H3 nDNA were insufficient to show the difference between these cryptic species. Although using concatenated genes it was not visualized, we suggest the need for future phylogenetic studies using other lines of evidence (such as larval morphology) and larger numbers of specimens to improve our knowledge of the natural relationships between this pair of species.

### *M. olfersii×M. faustinum*

Another fascinating result was the relative positions of *M. olfersii* and *M. faustinum*, which were located on separate branches on all phylogenetic analyses (Figs. 2, 3 and 5), but H3 (Fig. 4), which is too conservative gene. Distance analyses showed the percentage of intraspecific was lower than interspecific variation. *M. olfersii* data ranged from 0.00 to 0.18% for 16S, 0.00 to 0.95% for COI, and 0.00 to 0.27% for H3. The obtained values between *M. olfersii* and *M. faustinum* was 3.84% for 16S, ranging from 12.51 to 12.70% for COI and from 0.27 to 0.54% for H3. In previous study, *Macrobrachium* analyzed ranged 15.6% (*M. americanum* and *M. nattereri*) for 16S and 2.3 (*M. americanum* and *M. carcinus*) to 20.6% (*M. heterochirus* and *M. borelli*) for COI. In the same study it was showed an intraspecific divergence ranging from 0 to 0.9 for COI from different *M. olfersii* specimens [27].

This fact disagrees with previous morphological studies that suggested that *M. faustinum* is a junior synonym of *M. olfersii,* because of the close similarity between them [74], [39], [9], [11]. The voucher specimen that was deposited in GenBank as *M. faustinum* (ID: HM352461) was analyzed, and its identification is incorrect. Therefore, the sequence for “*M. faustinum*” available in GenBank should be redesignated as *M. olfersii.* This case proves the importance to verify the identification of the specimens before the molecular study and the necessity of the existence of voucher.

Our result is also supported by the geographical distributions of these species. *M. faustinum* occurs only in the West Indies and Florida [9], [75], [76], but the specimen available in GenBank was from Curarigua, Venezuela (IVIC 1083). *M. olfersii* does not occur in the West Indies [75].

Although the phylogenetic topology obtained here was well supported, another study has been conducted (Rossi & Mantelatto, in preparation) with larger numbers of specimens from some regions and including other related species, in order to complete the entire set of species and improve knowledge of the phylogenetic relationships of the *M. olfersii* complex.

### Gene Flow and Dispersion

Freshwater prawns of the genus *Macrobrachium* are thought to have originated from marine ancestors in the beginning of the Pleistocene, some of which subsequently migrated towards freshwater in more than one time [77], [64]. A colonization of freshwater is considered as the invasion by the ancestor of a lineage followed by the subsequent diversification of that lineage within continental waters [78]. These species acquired many physiological, ecological and behavioral adaptations [77], [79], [6]. Three basic types of larval developmental patterns can be recognized in this genus there are several species showing transitional developmental models. Some of them need estuarine and freshwater to complete their life cycle, which implicates in recurring migrations between both environments, such as *M. olfersii* [80], [6].

Molecular variation among the groups and the populations was not significant, again confirming the occurrence of a continuous gene flow. Consequently, the possibility of differentiation at the genetic level is decreased [81]. The existence of gene flow is plausible, since the *M. olfersii* larvae could be carried out on aquatic plants or associated with cultured species, as may have been the means of introduction of *M. olfersii* into Florida, United States [13]. On the other hand, the species may also have used favorable currents to reach Florida. This latter argument is strengthened by the finding of *M. olfersii* populations in all east-coast drainages of Florida [76].

Following the idea of *M. olfersii* to be undergoing a continuous process of adapting completely for freshwater environments [77], [32], [64], because it still depends on brackish water, Larvae of *Macrobrachium* can survive in high concentrations (over 30 ppt) of salt water [82], [83], [80]. *Macrobrachium olfersii* has been found in natural estuarine habitats at salinities up to 36 ppt. This finding indicates that the species has a higher salinity tolerance than was suggested by earlier laboratory studies [83], and it can survive in sea water for extend periods of time [76].

At the same time, studies of the physiology of freshwater shrimps have showed that although the Na^+^/K^+^ -ATPase systems of the animals do not function at maximal activity, the Na^+^ transport systems respond to salt loading when they are in the process of acclimating to high salinity [79]. We known that larvae of *Macrobrachium* species have a widely dispersion [11], [13], [83]. Marine current could carry *M. olfersii* larvae over long distances, from southern Brazil to the Caribbean or the contrary, allowing a continuous gene flow.

### Conclusions

In spite of its high morphological variability and wide geographic distribution, *Macrobrachium olfersii* has a lower intraspecific genetic divergence than interspecific. Phylogenetic analysis based on COI mtDNA sequences revealed surely that *M. olfersii* forms a monophyletic clade. Also, there were no differences among the populations. This result confirms that continuous gene flow exists among Caribbean and Brazilian populations of *M. olfersii*, as shown by the haplotype net, and supports the characterization of these populations as a single species. *Macrobrachium digueti* is a sister group, from the Pacific coast.

Moreover, the analysis of 16S mtDNA and H3 nDNA sequences provided evidence that *M. olfersii* has recently diverged from other *Macrobrachium* species from Central America, namely the *M. olfersii* complex. In conclusion, our findings confirm using H3 nDNA and 16S rDNA sequences as molecular markers for separated species identification whereas the COI gene is better suited to address genetic lineages and to explore possible cryptic species. However, addition of data from more specimens and other species is required to enhance the confirmation and resolution of the phylogenetic relationships of these groups. Morphological studies of the species belonging to this complex are in progress, to complete and elucidate this scenario.
